# Metabolic remodeling of glycerophospholipids acts as a signature of dulaglutide and liraglutide treatment in recent-onset type 2 diabetes mellitus

**DOI:** 10.3389/fendo.2022.1097612

**Published:** 2023-01-04

**Authors:** Juan Du, Liuqing Xi, Zhongxiao Zhang, Xiaoxu Ge, Wenyi Li, Wenfang Peng, Xiaohong Jiang, Wen Liu, Nan Zhao, Xingyun Wang, Xirong Guo, Shan Huang

**Affiliations:** ^1^Endocrinology Department, Tongren Hospital, Shanghai Jiao Tong University School of Medicine, Shanghai, China; ^2^Hongqiao International Institute of Medicine, Tongren Hospital, Shanghai Jiao Tong University School of Medicine, Shanghai, China

**Keywords:** type 2 diabetes mellitus, metabolic remodeling, dulaglutide and liraglutide, Glycerophospholipids, LC-MS

## Abstract

**Aims:**

As metabolic remodeling is a pathological characteristic in type 2 diabetes (T2D), we investigate the roles of newly developed long-acting glucagon-like peptide-1 receptor agonists (GLP-1RAs) such as dulaglutide and liraglutide on metabolic remodeling in patients with recent-onset T2D.

**Methods:**

We recruited 52 cases of T2D and 28 control cases in this study. In the patient with T2D, 39 cases received treatment with dulaglutide and 13 cases received treatment with liraglutide. Using untargeted metabolomics analysis with broad-spectrum LC-MS, we tracked serum metabolic changes of the patients from the beginning to the end of follow-up (12^th^ week).

**Results:**

We identified 198 metabolites that were differentially expressed in the patients with T2D, compared to the control group, in which 23 metabolites were significantly associated with fasting plasma glucose. Compared to pre-treatment, a total of 46 and 45 differentially regulated metabolites were identified after treatments with dulaglutide and liraglutide, respectively, in which the most differentially regulated metabolites belong to glycerophospholipids. Furthermore, a longitudinal integration analysis concurrent with diabetes case-control status revealed that metabolic pathways, such as the insulin resistance pathway and type 2 diabetes mellitus, were enriched after dulaglutide and liraglutide treatments. Proteins such as GLP-1R, GNAS, and GCG were speculated as potential targets of dulaglutide and liraglutide.

**Conclusions:**

In total, a metabolic change in lipids existed in the early stage of T2D was ameliorated after the treatments of GLP-1RAs. In addition to similar effects on improving glycemic control, remodeling of glycerophospholipid metabolism was identified as a signature of dulaglutide and liraglutide treatments.

## Introduction

The rapid rise of type 2 diabetes mellitus (T2D) poses one of the greatest threats to global health. Diabetes was diagnosed in approximately 463 million adults aged 20 to 79 in 2019, according to the International Diabetes Federation. Furthermore, it is estimated that in 2045, the number of individuals with T2D will reach 700.2 million (1, 2). Obesity is generally considered to be a strong risk factor for diabetes (3). Overall, about 80% of people with T2D are overweight or obese. In the obese state, excessive accumulation of visceral fat causes adipose tissue dysfunction that strongly contributes to the development of obesity-related comorbidities (3, 4).It is one of the most important public health challenges in the world to tackle the dual epidemic of T2D and obesity, as both are associated with increased mortality and morbidity (5). Glucagon-like peptide-1 receptor agonists (GLP-1RAs) have been used to treat T2D and obesity for the past 10 years. A single GLP-RA, liraglutide, has been approved for treating obesity in people with type 2 diabetes because it reduces body weight (6). Furthermore, the cardiovascular benefits of GLP-1RAs have led to significant progress in the management of diabetes (7). A greater effect on fasting glucose has been observed with long-acting GLP-1RAs, such as dulaglutide and liraglutide, compared with short-acting GLP-1RAs, mediated through insulinotropic and glucagonostatic actions. A head-to-head clinical trial found that once-weekly dulaglutide had a similar safety and tolerability profile to once-daily liraglutide for the reduction of HbA1c (6, 8). Despite these successes, less is known about the benefits of metabolic remodeling with the treatment of GLP-1RAs, in addition to improved glycemic control and weight loss.

Both humans and animal models have been examined for metabolic changes associated with T2D and obesity (9–11). Obese and diabetic individuals have both been found to have high levels of branched-chain amino acids (BCAAs) (leucine, isoleucine, and valine), as well as some of their tissue metabolites, while glutamine and glycine levels are decreased (12, 13). Other non-protein nitrogen compounds, such as uridine and uric acid, were significantly correlated with insulin resistance in ob. Insulin resistance was significantly associated with other nitrogen compounds other than protein, such as nucleotides and nucleosides, as well as their metabolites, namely uridine and uric acid in obese subjects (14, 15). Given the changes in serum metabolites that exist before the onset of metabolic disease, longitudinal studies suggest that BCAA and hyocholic acid species may serve as novel biomarkers for the early detection and differential diagnosis of metabolic syndrome (16, 17). Metabolomics has been identified as a useful tool for evaluating the metabolic effects of glucoregulatory and weight loss-promoting drugs, including GLP-1RAs. Despite the non-inferiority of a once-weekly GLP-1RA dulaglutide agonist to once-daily liraglutide, knowledge of the metabolic differences in response to different long-acting GLP-1RAs is still sparse, as previous study only reported the modulation of gut microbiome following the treatment with GLP-1RAs in the animal model (18). A high-resolution landscape of metabolites during GLP-1RAs treatment is still poorly understood. Therefore, to investigate the metabolic remodeling in relation to GLP-1RAs, we were motivated to perform the metabolomics analysis in recent-onset T2D individuals with GLP-1RAs treatment.

Here, 52 recent-onset T2D cases and 28 control cases were recruited. We first identified a few metabolites related to T2D and metabolic pathways that offer a comprehensive view of the metabolite changes in T2D. In patients with T2D, 39 cases received dulaglutide treatment (once-weekly, 1.5 mg, DU group) and 13 cases received liraglutide treatment (once-daily, 1.8 mg, LIGA group), respectively. We then determined the effects of pharmacologically stimulated GLP-1 receptor function on metabolic remodeling, and the differences in improved metabolism during 12-week treatment by longitudinal analysis. Lastly, we integrated the longitudinal analysis with the case-control status of diabetes to uncover metabolic pathways and potential targets associated with GLP-1RAs.

## Materials and methods

### Study design and participants

The specialist inpatients were adults (≥ 18 years) with BMI ≥ 24 kg/m^2^, and newly diagnosed with T2D (known duration of diabetes less than 1 month). The diagnosis of T2D was based on WHO criteria. All subjects went through three periods: intensive insulin therapy phase (1 week), switching to GLP-1RAs (at baseline), and follow-up (12 weeks). Participants in the study were interviewed between September 15, 2021, and February 30, 2021. The criteria for short-term continuous subcutaneous insulin infusion (CSII) included HbA1c ≥ 9.0% or fasting blood glucose ≥ 11.1 mmol/L or patients with obvious symptoms of hyperglycemia. We assigned participants to DU or LIGA group according to the patient’s personal choice. Exclusion criteria included the use of other anti-hyperglycemic drugs, the clearance of creatinine less than 90 mL/min, or a history of pancreatitis or gastrointestinal surgery.

Twenty-eight lean, metabolically healthy controls (CTL, 17 male, 11 female; age 45.18 ± 11.48 SD; BMI 22.78 ± 1.31 SD) were enrolled in the clinics of Tongren Hospital. Criteria for “metabolically healthy” are as follows: no previous history of hypertension and systolic blood pressure (SBP)/diastolic blood pressure (DBP) < 140/90 mmHg; fasting blood glucose (FBG) < 6.1 mmol/L, OGTT (2 h) <7.8 mmol/L and no previous history of diabetes; no previous history of high cholesterol (total cholesterol (TC) <5.18 mmol/L) and fasting serum high-density lipoproteincholesterol (HDL-c) ≥ 0.9 mmol/L (men) or ≥1.0 mmol/L (women) and fasting plasma TG <1.7 mmol/L; no history of cardiovascular or endocrine disease.

the Internal Review and Ethics Boards of Tongren Hospital, which is affiliated with Shanghai Jiao Tong University, has approved this clinical study (No. 2021-059-01); informed consent was obtained from all patients. Good clinical practices and country-specific requirements were followed according to the Declaration of Helsinki.

### Clinical measurements

We assessed the following characteristics of the participants: sex, region, age, body weight, height, waist circumference, blood pressure, and body mass index (BMI). Plasma samples were collected from participants after a fast of 8 to 12 hours. An automatic biochemical analyzer was used to determine biochemical indexes, including FBG, HbA1c, alanine aminotransferase (ALT), aspartate aminotransferase (AST), TC, TG, low-density lipoprotein cholesterol (LDL-C), HDL-C and creatinine (CR) (AU5800 clinical chemistry analyzer; Beckman Coulter Inc., Brea, CA, USA), Serum insulin and c-peptide were measured by radioimmunoassay. Based on all analyses, there was 6% intra-assay variation and 10% inter-assay variation. The Homeostasis Model Assessment (HOMA2) Estimates steady state beta cell function (%β) and insulin resistance index (IR), as measured by the HOMA2 Calculator (https://www.dtu.ox.ac.uk/homacalculator/).

### Sample collection and preparation

The fasting serum samples were collected from 80 participants before treatment (52 newly diagnosed T2D cases and 28 controls). Thirty-one of them (25 cases out of 39 samples in the DU group and 8 cases out of 13 samples in the LIGA group) provided serum samples for metabolomics after 12-week treatment. The fasting serum samples were remained after clinical indexes determination and immediately stored at -80 °C. The serum was vortexed for 2 minutes after being added to 300 µl of methanol (containing a standard of 5 g/ml L-2-chlorophenylalanine). A 200-µl supernatant was obtained after centrifugation at 13,000 rpm at 4 °C for 10 minutes. In order to prepare the quality control samples, the same volume of serum was taken from all samples and mixed evenly.

### Untargeted metabolomics by LC–MS

A UPLC system equipped with an electrospray ionization source was used for non-targeted metabolomics profiling of serum samples. A data dependent (dd-MS2, Top N=10) MS/MS mode was used with a full scan mass resolution of 17,000 at an m/z of 200. A range of 100 to 1,500 was scanned. A brief description of the chromatographic conditions is given below: the injection volume was 2 µl, the column temperature was 25 °C, the flow rate was 0.35 ml/min, and the mobile phase was liquid aqueous solution containing 0.1% formic acid, and liquid b-acetonitrile containing 0.1% formic acid. Optimal chromatographic gradients were: 0-2 min, 5% in liquid B; 2-10 min, 5-95% in liquid B; 10-15 min, 95% in liquid B; 15-18 min, 5% in liquid B. Thermo Xcalibur 2.2 software was used to acquire the data (Thermo Scientific, San Jose, USA).

Compound Discoverer software (Thermo Fisher Scientific) was used to align and extract peaks. After obtaining the retention time, m/z, and peak area information, we obtained a data table. In order to analyze the data, we used Simca-P software version 13.0 (Umetrics, Umea, Sweden) to perform a principal component analysis (PCA) and an analysis of partial least squares discrimination (PLSDA). An unsupervised PCA analysis was used to evaluate the overall trend in segregation between these samples. We normalized and Pareto-scaled the ion peaks. A quality assessment was performed using R2Y(cum) and Q2(cum) parameters for the PLS-DA models generated. Significant differences in metabolites between the T2D and control groups were detected using a supervised PLSDA analysis model. Variables with variable importance in the projection (VIP) values >1.0 were selected based on the PLS-DA model, and a two-tailed Student’s t-test by SPSS Statistics 18.0.0 was used to calculate p value, and p value < 0.05 was considered statistically significant. Using partial correlation analysis, metabolic traits of the disease (age, BMI, blood pressure, glucose/insulin/HOMA-%β) were determined that exhibit the strongest association with metabolites showing significantly different levels between the diseases. A standard metabolite library was used to identify metabolites, matching their exact mass, fragment ion mass, and retention time. The online processing tool of metabolomics data was used to analyze metabolic pathways to further predict the molecular mechanism of dulaglutide and liraglutide for the treatment of GLP-1RAs. MetaboAnalyst 4.0 (http://www.metaboanalyst.ca/) and Genecards (https://www.genecards.org/) were used to identify and beneficiate affected metabolic pathways. An interaction network between compound-protein-pathway was developed to explain the functional mechanism. The enriched metabolic pathways were merged with Genecards above by Pharm mapper to produce a virtual predictive target through MetaboAnalyst 4.0 (http://www.lilab-ecust.cn/pharmmapper/). In order to estimate the importance of each node, we used two well-established measures, degree and betweenness centrality, and STRING exported all these parameters (https://www.string-db.org/).

### Statistical analysis

A statistical analysis was conducted using IBM SPSS 25 and Thermo Xcalibur 2.2 software. (Thermo Scientific, San Jose, USA). A normal distribution was ensured by log-transforming or taking the square root of skewed variables. As appropriate, comparisons were conducted using t-tests, Wilcoxon-Mann-Whitney tests, and one-way ANOVAs. Significance was defined as p ≤ 0.05. For comparing ordinal and non-normal variables, non-parametric tests were used.

## Results

### The characteristics of study subjects and the research design

**Figure 1:**
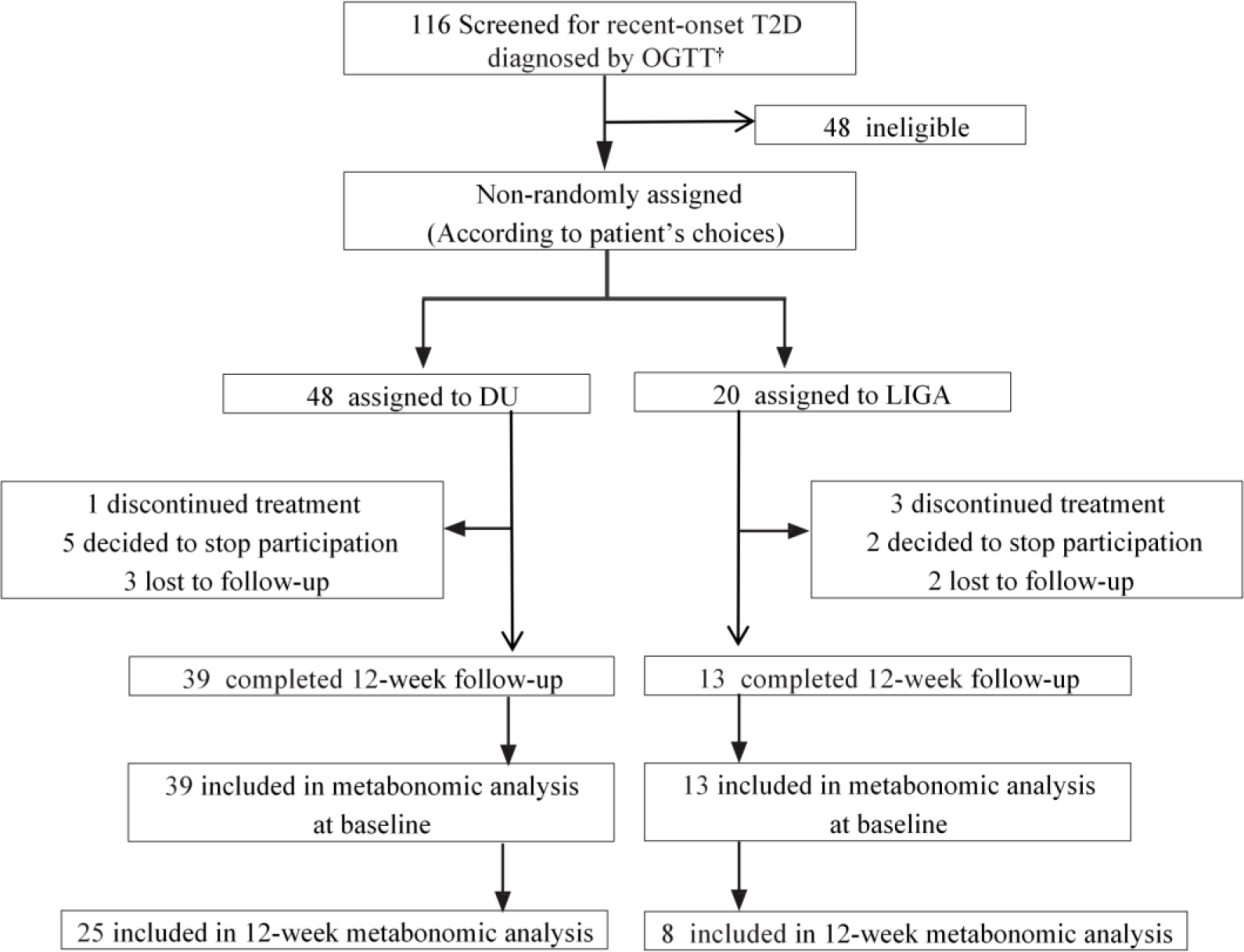
The trial profile of this study. ^†^ Data were collected from September 15, 2021, to November 10, 2021. T2D, type 2 diabetes; OGTT, oral glucose tolerance test; DU, dulaglutide; LIGA, liraglutide.

**Figure 2:**
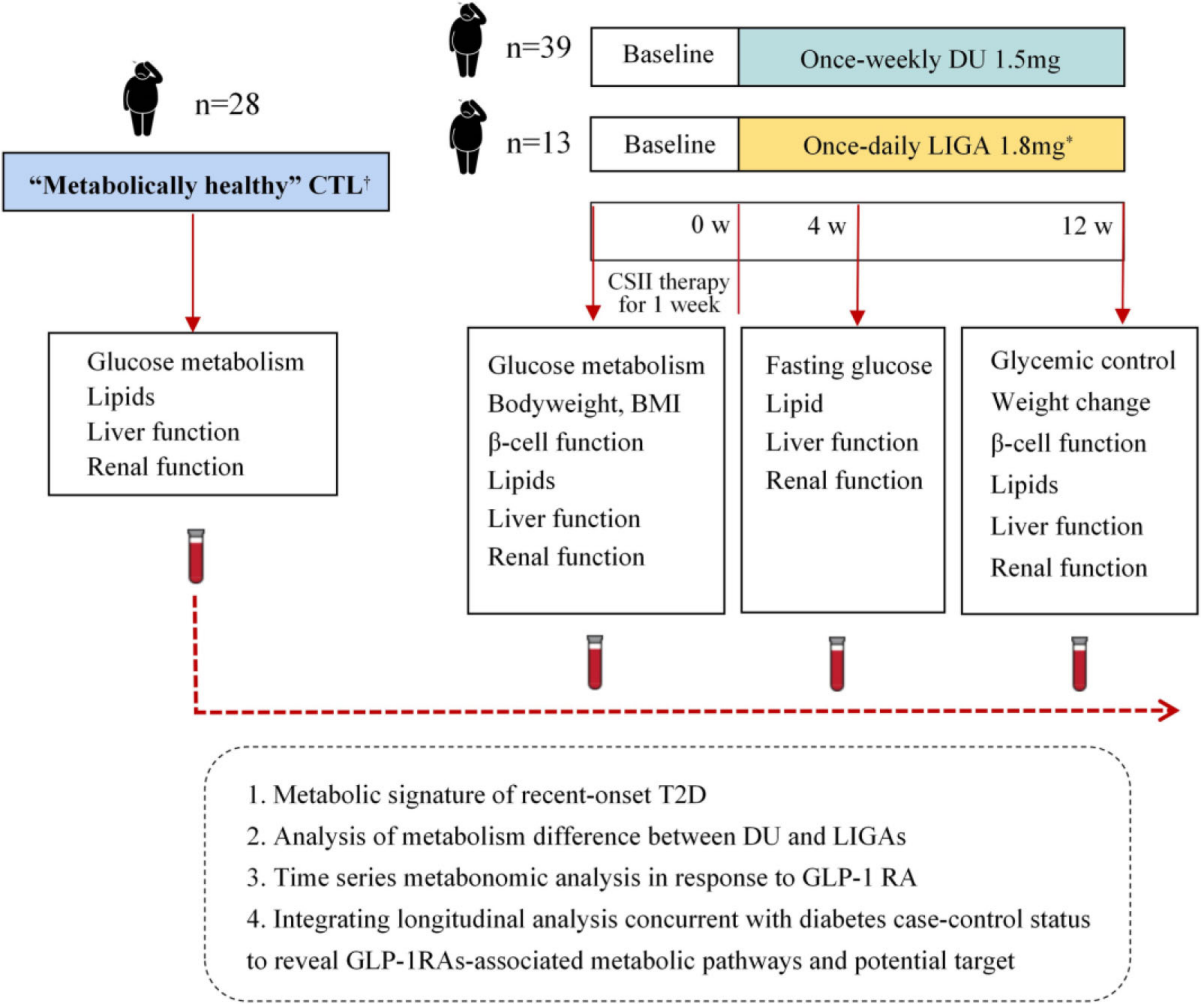
Study design and comparative analysis of metabolic profiles. *Liraglutide was up titrated from 0.6 mg/day in the first week, to 1.2 mg/day in the second week, and then to 1.8 mg/day in the third week. ^†^ Criteria for “metabolically healthy” are as follows: no previous history of high blood pressure and systolic blood pressure (SBP)/diastolic blood pressure (DBP) < 140/90 mmHg; no previous history of diabetes and fasting blood glucose (FBG) < 6.1 mmol/L, OGTT (2 h) < 7.8 mmol/L and; no previous history of high cholesterol (total cholesterol (TC) < 5.18 mmol/L) and fasting plasma TG < 1.7 mmol/L and fasting serum high-density lipoproteincholesterol (HDL-c) ≥ 0.9 mmol/L (men) or ≥ 1.0 mmol/L (women); no history of cardiovascular or endocrine disease. DU, dulaglutide; LIGA, liraglutide; CTL: control; CSII, continuous-subcutaneous insulin infusion; BMI, body mass index; CGMS, continuous glucose monitoring system; GLP-1 RA, glucagon-like peptide-1 receptor agonist.

**Figure 3:**
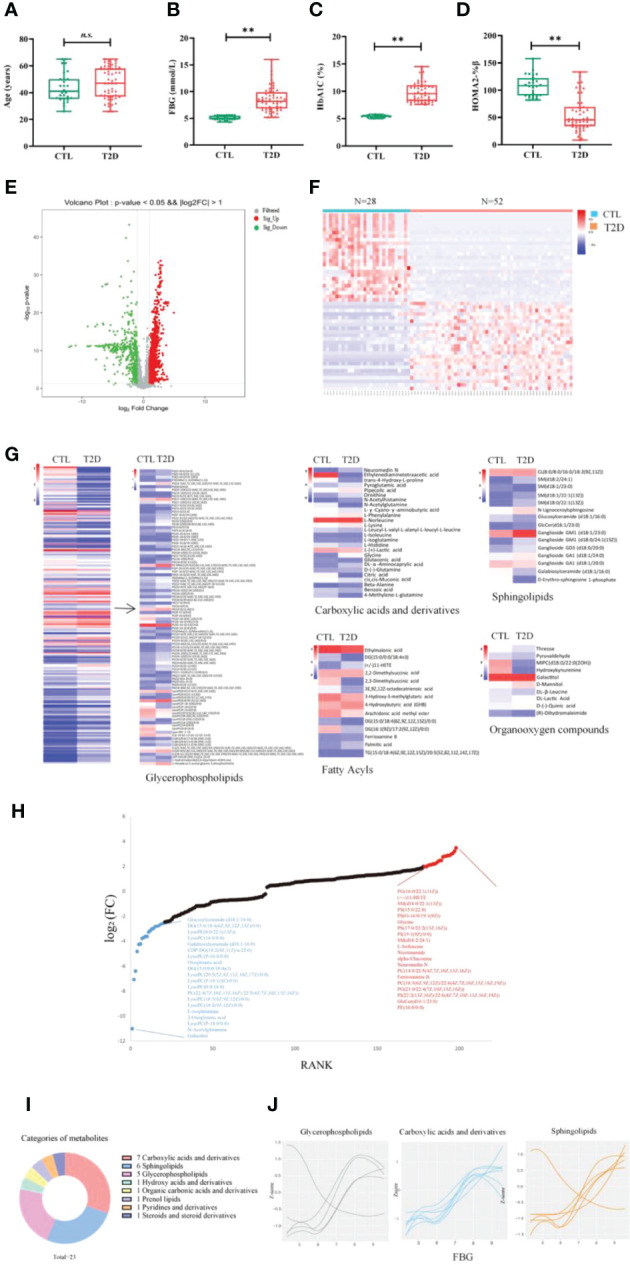
Change in metabolic signatures and metabolite groups at the early stage of diabetes development. The levels of age **(A)**, FBG **(B)**, HbA1c **(C)** and HOMA2%β **(D)** in controls (n = 28) and in patients with T2D at baseline (n = 52). Differences between the T2D group and the CTL group were tested by independent sample t test (normally distributed variables) **p < 0.01. n.s. the difference is not statistically significant. **(E)** Volcano plot showing -log_10_ p-value against log_2_-fold change of 198 metabolites in T2D cases versus controls. **(F)** Hierarchical cluster analysis of differential metabolites in T2D at baseline, compared to the CTL group. **(G)** Comparison of the differential metabolites between the T2D and CTL groups. Fold changes are log transformed and indicated by color scale in matrix. **(H)** Plot shows the top 15 increased (red) and decreased (blue) metabolites in T2D. **(I)** The categories of the 23 metabolites significantly associated with FBG (correlation coefficient >0.7). **(J)** The values of three main categories of FBG-associated metabolites are plotted with a Loess curve against the FBG. FBG, fasting blood glucose; HbA1c, glycated hemoglobin; HOMA2%β, the Homeostasis Model Assessment (HOMA) estimates steady state beta cell function (%β).

The study recruited 52 recent-onset T2D cases and 28 control cases. In total, 39 incident T2D cases treated with once-weekly dulaglutide 1.5 mg (DU group) and 13 cases treated with once-daily liraglutide 1.8 mg (LIGA group), and completed a 12-week treatment (**Figure 1**). The detailed information used for the demographic and clinical analysis is listed in **Figure 2**. Serial blood samples from pre-treatment, 4-week and 12-week post-treatment were collected and profiled for metabolomics. The recent-onset T2D group was made up of individuals with an age range of 28 to 65 (the median was 47, the interquartile range was 37 to 58) years old, a BMI of 28.78 ± 3.92 kg/m^2^. Among them, 63.50% were male. Neither age nor race/ethnicity were significantly different (**Table S1** and **Figure 3A**). The detailed demographic information and the baseline metabolic characteristics of the participants were listed in Table S1. In the T2D group, the BMI (p = 0.001), FBG (p = 0.001), and HbA1c (p = 0.001) were higher than in the CTL group (**Table S1** and **Figures 3B, C**), whereas HOMA2-%β was significantly decreased in T2D patients (**Figure 3D**). Individuals with T2D had higher blood pressure (p= 0.001), TG (p = 0.003), TC (p = 0.003), and LDL-c (p = 0.001) in comparison to controls (**Table S1**).

A total of 1,147 metabolites were included in the final dataset after data curation and annotation. The dataset was used to i) characterize the differentiation of metabolite profiles and the alteration of metabolite groups between the recent-onset T2D cases and metabolically healthy controls, ii) shape and present a trajectory of metabolic remodeling during GLP-1RA treatment by tracking metabolic changes across individuals, and determined the universal and differential response to dulaglutide and liraglutide by longitudinal analysis, iii) identify metabolic pathways and potential targets associated with GLP-1RAs in follow-up samples concurrent with diabetes case-control status.

### The modulation of metabolic signatures and metabolites in the early stage of diabetes development

The metabolic profiles of participants with recent-onset T2D and those of CTLs at baseline are well separated by partial least squares-discriminant analysis (PLS-DA), suggesting significant metabolic changes in T2D compared to participants without T2D (**Figures S2A–D**). The differences in metabolite expression between T2D cases and controls were statistically significant (p value less than 0.05) for 198 metabolites, including 82 up-regulated and 116 down-regulated metabolites (the top 50 differentially regulated metabolites are shown in **Table S2**). The differential metabolites were visualized in the volcano plot (**Figure 3E**). The Spearman correlation analysis presented in the heat map showed that there was a significant correlation of all differential metabolites in the DU or LIGA treatment group, compared to the CTL group (**Figure 3F**). More than half of 15 most upregulated metabolites in individuals with recent-onset T2D belong to glycerophospholipids (p value < 0.05), while the downregulated metabolites (N = 15) were mainly lipids or lipid-like molecules, such as lysophosphatidylcholines (lysoPC) and fatty acyls (**Figure 3H**).

The classes of 198 differentially regulated metabolites included carboxylic acids and derivatives (BCAAs, glycine and organic acids), lipids (phospholipids and sphingolipids, fatty acyls), and organooxygen compounds (**Figure 3H**), which were previously reported in the T2D cohorts (general adult population). Particularly, 94 glycerophospholipids showed differential regulation between the T2D and CTL groups, in which the majority of lysoPC (except for LysoPC a 16:1) were elevated in the T2D group, as well as the PC aa C group (except for PC aa C11:1 and C22:4) (**Figure 3G** and **Table S2**). It is interesting to note that amino acids (AAs) were detected both in up-regulated and down-regulated sets. Eight AAs (glutamine, alanine, glycine, isoleucine, ornithine, phenylalanine, norleucine and lysine) were up-regulated, with acetylglutamine, 4-Methylene-L-glutamine, isoglutamine, histidine being down-regulated. (**Figure 3G** and **Table S2**). Our analysis identified 23 metabolites that were significantly associated with FBG between the two cohorts. This set of 23 metabolites associated with FBG were primarily carboxylic acids and derivatives (n = 7), sphingolipids (n = 6), and phosphatidylcholines (n = 5) (**Figures 3I, J**). Glycine was the most significantly associated metabolite (Pearson correlation coefficient 0.91, p value 0.05).

### Analysis of cross-sections at follow-up: The metabolome changes in response to GLP-1 RAs and the implications for glycerophospholipid metabolism remodeling

**Figure 4:**
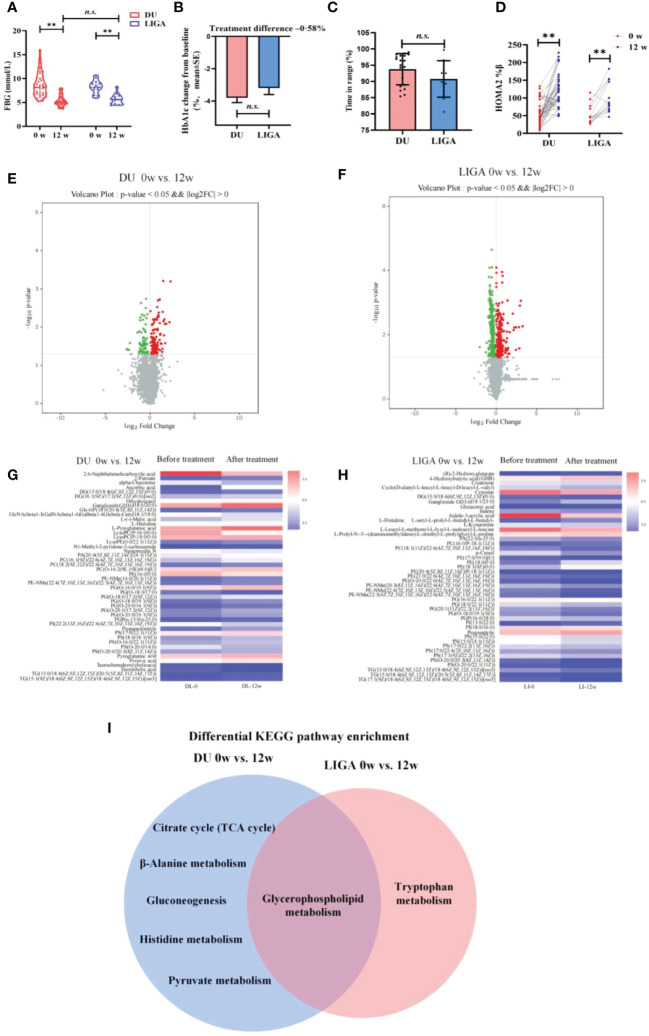
Cross-sectional analysis of metabolome changes in response to GLP-1RAs and implications for glycerophospholipid metabolism remodeling. **(A)** The levels of FBG at baseline and 12 weeks after treatment. **(B)** The decreasing levels of HbA1c after treatment with DU and LIGA at baseline and 12^th^ weeks. **(C)** Comparison of the quality of the glucose control, as evaluated by the percentage of time in the range of 8.0 to 10.0mmol/L. **(D)** Change in HOMA2%β index after treatment with DU and LIGA at baseline and 12^th^ week. **(E)** Volcano plot of -log_10_ p-value against log_2_-fold change of 46 metabolites in the DU group at baseline and 12^th^ week. **(F)** Volcano plot of -log_10_ p-value against log_2_-fold change of 45 metabolites in LIGA group at baseline and 12^th^ week. **(G, H)** Comparison of differential metabolites before and after treatments in the DU and LIGA groups. Fold changes are log transformed and indicated by color scale in matrix. **(I)** Venn diagram depicting KEGG pathway analysis of differential metabolites in the DU and LIGA groups. Differences between T2D and CTL were tested by independent sample t test (normally distributed variables). **p < 0.01. n.s. the difference is not statistically significant. BMI, body mass index; FBG, fasting blood glucose; HbA1c, glycated hemoglobin; DU, dulaglutide; LIGA, liraglutide; HOMA2%β, the Homeostasis Model Assessment (HOMA) estimates steady state beta cell function (%β).

Post-treatment samples (25 cases out of 39 samples in the DU group) were used to gain further insight into the metabolic changes associated with GLP-1RAs, while 8 cases out of 13 samples in the LIGA group) were further analyzed cross-sectionally to identify the GLP-1RAs-associated metabolites. Age, BMI, FBG, and HbA1c at baseline did not differ significantly between the groups (**Figures S3A, B, E, F**), as well as other key clinical characteristics, including blood pressure, liver function, kidney function, and blood lipids (**Figure S3**). Compared to baseline, participants in the DU and LIGA groups after 12 weeks of follow-up showed significantly lower levels of FBG, 2-hour postprandial blood glucose, body weight, and HbA1c (**Figures 4A, B**, **S4A**, **C**). The glucose metric analysis in both groups was calculated using CGMS data (n=20 in the DU group, n=10 in the LIGA group) during 4 weeks of treatment. The quality of the glucose control, as evaluated by the percentage of time in the range of 8.0 to 10.0mmol/L, did not differ statistically between the DU group and the LIGA group (**Figure 4C**). An increment in HOMA2%β after 12 weeks of treatment was observed in both groups (**Figure 4D**). Improvements in glycemic control, insulin resistance, and body weight were similar in two groups (**Figures 4A**, **S4A–D**).

Furthermore, in both the DU and LIGA groups, the well-separated metabolic profiles before and after treatments (4 weeks or 12 weeks of treatment) in the PLS-DA score plot indicate the significant effect of GLP-1RAs on metabolomics (**Figures S5A–D**). Differential metabolites were visualized in the volcano plot (**Figures 4E, F**). After 12 weeks, the DU and LIGA groups had 46 and 45 differentially regulated metabolites, respectively (log2FC >1.2, p value<0.05) (**Figures 4G, H**). In order to gain a deeper understanding of T2D metabolic pathways, all differential metabolites were analyzed using the KEGG pathway analysis. As indicated by KEGG pathway analysis, glycerophospholipid metabolism was profoundly affected in both groups (p value = 0.001) (**Figures 4I**, **S5E, F**), suggesting its important role in association with dulaglutide and liraglutide treatments. For dulaglutide treatment, other metabolic pathways, including the citrate cycle (TCA cycle), β-alanine metabolism, glycolysis, histidine metabolism, and pyruvate metabolism, were also enriched.

### Integrating longitudinal analysis with case-control diabetes status to identify metabolic pathways associated with GLP-1RAs

**Figure 5:**
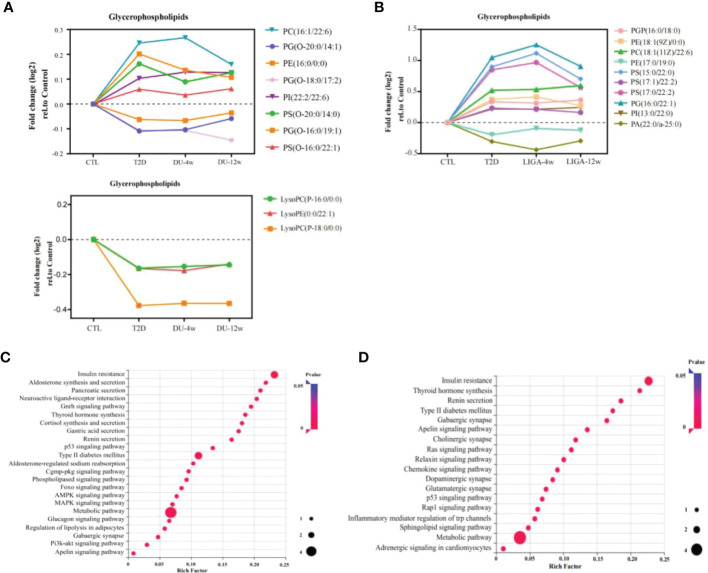
Integrating longitudinal analysis concurrent with diabetes case-control status to reveal GLP-1RAs-associated metabolic pathways and potential targets. **(A, B)** Longitudinal trajectories of significant metabolites involved in glycerophospholipid metabolism in response to DU and LIGA, respectively. **(C, D)** Regulated pathways associated with DU **(C)** and LIGA **(D)**. DU, dulaglutide; LIGA, liraglutide.

To further examine metabolite dynamics and illuminate metabolic pathways, we traced the dynamic changes of metabolites during 12-week treatment in each group. All differentially expressed metabolites detected in the DU group and the LIGA group compared to the baseline were listed in **Tables 1**, **2**, respectively. In the DU group, PC (16:1/22:6), PE (16:0/0:0), PS(O-20;0/14:0) were elevated before treatment, but showed a decrease pattern after 4 weeks and 12 weeks of treatment. On the contrary, lysoPC (P-16:0, P-18:0) and lysoPE (22:1) showed a greater decrease in T2D cases but increased after 12 weeks of treatments (an increase of 15%) (**Figure 5A**). Similar trends were observed in the LIGA group, such as PE (18:1/0:0), PS (16:0/22:1), PS (15:0/22:0) and PS (17:1/22:2) showed a greater decrease in T2D cases but increased after treatments (**Figure 5B**). Notably, like our findings from the cross-sectional analysis after treatment, most differentially regulated metabolites identified by longitudinal analysis also belong to glycerophospholipids, which emphasizes the association of remodeling glycerophospholipid metabolism and GLP-1RAs treatments. Our results suggested that there was a metabolic dysmetabolism of lipids present in the baseline status of the patients with newly onset T2D, which ameliorated partially during GLP-1RAs treatments. In the DU group, differential metabolites, such as the carboxylic acids and derivatives (e.g., Neuromedin N and L-Histidine), sphingolipids (e.g., TG (15:1/18:4) and Glc-GP (18:0/20:4) were also altered in response to 12 weeks of dulaglutide treatment (**Figure S6A**). Additionally, 4-hydroxybutyric acid (GHB) increased at baseline, but decreased at follow-up in the LIGA group (**Figure S6B**).

**Table 1:** The longitudinal trajectories of 22 significant metabolites in response to dulaglutide overlapped with those of T2D at baseline.

Name	Class
PG(O-20:0/14:1(9Z))	Glycerophospholipids
PE(16:0/0:0)	Glycerophospholipids
PG(O-18:0/17:2(9Z,12Z))	Glycerophospholipids
LysoPC(P-16:0/0:0)	Glycerophospholipids
PI(22:2(13Z,16Z)/22:6(4Z,7Z,10Z,13Z,16Z,19Z))	Glycerophospholipids
LysoPC(P-18:0/0:0)	Glycerophospholipids
LysoPE(0:0/22:1(13Z))	Glycerophospholipids
PC(16:1(9Z)/22:6(4Z,7Z,10Z,13Z,16Z,19Z))	Glycerophospholipids
PS(O-20:0/14:0)	Glycerophospholipids
PG(O-16:0/19:1(9Z))	Glycerophospholipids
PS(O-16:0/22:1(11Z))	Glycerophospholipids
Neuromedin N	Carboxylic acids and derivatives
L-Histidine	Carboxylic acids and derivatives
Pyroglutamic acid	Carboxylic acids and derivatives
DG(16:1(9Z)/17:2(9Z,12Z)/0:0)	Fatty Acyls
TG(15:1(9Z)/18:4(6Z,9Z,12Z,15Z)/18:4(6Z,9Z,12Z,15Z))	Glycerolipids
Glc-GP(18:0/20:4(5Z,8Z,11Z,14Z))	Glycerolipids
alpha-Chaconine	Steroids and steroid derivatives
Ganglioside GD3 (d18:0/20:0)	Sphingolipids
L-(-)-Malic acid	Hydroxy acids and derivatives
2-Furoate	Furans
Dihydrouracil	Diazines

**Table 2:** The longitudinal trajectories of 14 significant metabolites in response to liraglutide overlapped with those of T2D at baseline.

Name	Class
PGP(16:0/18:0)	Glycerophospholipids
PC(18:1(11Z)/22:6(4Z,7Z,10Z,13Z,16Z,19Z))	Glycerophospholipids
PE(17:0/19:0)	Glycerophospholipids
PS(15:0/22:0)	Glycerophospholipids
PS(17:1(9Z)/22:2(13Z,16Z))	Glycerophospholipids
PS(17:0/22:2(13Z,16Z))	Glycerophospholipids
PG(16:0/22:1(11Z))	Glycerophospholipids
PI(13:0/22:0)	Glycerophospholipids
PA(22:0/a-25:0)	Glycerophospholipids
Glutaconic acid	Carboxylic acids and derivatives
PE(18:1(9Z)/0:0)	Carboxylic acids and derivatives
4-Hydroxybutyric acid (GHB)	Fatty Acyls
Cytosine	Diazines
(R)-2-Hydroxyglutarate	Hydroxy acids and derivatives

Compound-protein-pathway interactions were established to better understand metabolite pathways and their metabolic processes. Significant (p value < 0.05) pathways were chosen as potential targets. In particular, the insulin resistance pathway (p value < 0.05) and type 2 diabetes mellitus (p value < 0.05) were found to be enriched in both the DU and LIGA groups (**Figures 5C, D**). Multiple pathways involved in energy metabolism are expressed in the DU group, including apelin metabolism, phospholipase signaling pathway, Foxo signaling pathway, glucagon signaling pathway, and regulation of lipolysis in adipocytes. The pathways with p value < 0.05 in the LIGA group were associated with apelin metabolism and the sphingolipid signaling pathway. Interestingly, the DU group was significantly more enriched in signals related to aldosterone synthesis, thyroid hormone production, and cortisol production, which may suggest a possible correlation with regulation of endocrine hormones. In the LIGA group, the finding of adrenergic signaling in cardiomyocyte metabolism indicated the role of LIGA in cardiovascular disease.

Further, the topological parameter was used to investigate the core network integrating metabolites and proteins in order to identify significant proteins that correlate with the metabolites found in the study. In the core network, all nodes had a degree greater than 3. **Table 3** shows that these proteins may be potential targets for dulaglutide or liraglutide. For example, GLP1R1 and GNAS proteins were related to insulin resistance. These potential targets provided us with some clues about the metabolic mechanism of GLP-1RAs in the treatment of T2D.

**Table 3:** The proteins of key protein-metab1ite network in the DU and LIGA groups.

DU group			LIGA group		
No.	Protein	Degree	No.	Protein	Degree
1	GLP1R	3.5759	1	GLP1R	3.5604
2	ARRB1	3.7672	2	GNAS	3.7517
3	GNAS	3.3513	3	LEG3	3.3358
4	GNGT1	3.0886	4	EGFR	3.0731
5	GCG	3.6718	5	TAP1	3.6563
6	GSR	3.9392	6	AMPK	3.9237
7	DCK	3.1178	7	HXK4	3.1023
8	FIBG	3.8304	8	GMPR1	3.8149
9	MAPK	3.3545	9	FKB1A	3.339
10	GSTT2	3.5978	10	AMY1A	3.5823
11	ABL1	3.4052	11	CCL5	3.3897
12	IMPA1	3.1767	12	Arl5b	3.1612
13	RAN	3.3574	13	BCAT2	3.3419
14	EGFR	3.5094	14	KTHY	3.4939
15	APAF	3.6077	15	AKT1	3.5922
16	GMPR2	3.5722	16	HINT1	3.5567
17	CHIT1	3.5137	17	HXK1	3.4982
18	ITPKA	3.9462	18	P07741	3.9307
19	ADK	3.0662	19	P06737	3.0507
20	DAPK1	3.0916	20	CBR1	3.0761

DU, dulaglutide; LIGA, liraglutide.

## Discussion

In our study, we offered a comprehensive view of the metabolite changes in T2D. Then we determined the effects of pharmacologically stimulated GLP-1 receptor function on metabolic remodeling and compared the differences in improved metabolism during 12-week treatment by longitudinal analysis. This is the first study to implement metabolomics to comprehensively explore the dynamic metabolic responses of T2D patients treated with GLP-1RAs, and to distinguish the differences in the metabolic remodeling of two GLP-1RA drugs (i.e., dulaglutide and liraglutide).

Compared to metabolically healthy subjects with normal weight, 198 differentially regulated metabolites were identified in T2D cases, and the major classes including glycerophospholipids, carboxylic acids, and derivatives (i.e., BCAAs, glycine, and organic acids), were significantly regulated (**Figures 3F, G**). Increasing evidence from human and animal studies indicates that a number of metabolites could contribute to specific metabolic disturbances in the pathogenesis of metabolic syndrome (e.g., glucose tolerance, obesity, insulin resistance, and NAFLD) (19, 20). The level of BCAAs among adults increased before the onset of T2D, which could impair pancreatic beta cell function and affect insulin signaling (21, 22). An analysis of multi-metabolite signatures of T2D revealed altered metabolic fingerprints associated with the metabolism of amino acids, carbohydrates, and microbiota metabolism (23). Many of the T2D-associated metabolites (e.g., glycerophospholipids, sphingolipids, and amino acids), which were revealed in other studies, were also validated in our study (24, 25). Among the altered metabolites in the T2D cases, the major class was glycerophospholipids. In addition, a small group of glycerophospholipids was also identified for their association with the treatment of dulaglutide or liraglutide. Glycerophospholipid perturbance has been linked to the pathogenesis of diabetes in animal studies, as well as in prospective studies in humans (26). Our findings are in line with the known relationships between glycerophospholipid disturbance and the pathogenesis of diabetes. Recently, in a prospective study, eight glycerophospholipids, especially PCs, were associated with the *de novo* lipogenesis pathway and positively correlated with incident diabetes among Asians (27).

Compared to the patients with CTL, patients with recent-onset T2D had significantly higher levels of PC, whereas, surprisingly, the concentrations of lysoPC were lower in the T2D group. In a recent study, serum lysoPCs were associated with a reduced risk of type 2 diabetes (28). Previous studies found that lysoPC levels were lower in individuals with obesity, insulin resistance, and T2D (29–31). These findings suggest that the dynamic alterations of lysoPCs may potentially be associated with the pharmacological actions of GLP-1 receptor signaling by dulaglutide and liraglutide on glucose metabolism and lipid handling in T2D patients. Serum LysoPCs are primarily generated by secretory or lipoprotein-bound phospholipase A2 from lipoproteins or membrane-derived PCs. A second source of plasma lysoPC is endothelial lipase, which also creates HDL and oxidized LDL by secreting lecithin-cholesterol acyltransferase from the liver. On the other hand, lyso-PCs can also be reacylated into PC by lysoPC acyltransferase. Previous studies have reported that both lysoPCs and lysoPEs, grouped as LPs, were associated with a reduced risk of T2D (32). In adipocytes, lysoPC(C16:0) enhanced glucose uptake in an insulin-independent and protein kinase C-dependent manner (33). These findings provide evidence of the beneficial effects of lysoPC in metabolic diseases *in vivo* and *in vitro*. Furthermore, A significant change in amino acid metabolism occurred during the pathogenesis of T2D, in which some amino acids served as substrates for gluconeogenesis. It is worth noting that the metabolic pathways associated with T2D differed between studies due to the different inclusion criteria, heterogeneity regions, sample sizes, or analytical methods.

It is the first study to determine the trajectory changes of the metabolites in the cohorts after treatment with different GLP-1RAs. Both dulaglutide and liraglutide are widely advocated as means of improving glycemic control outcomes, but little is known about the metabolic remodeling in serum responsible for its beneficial effects. The metabolic profiles here revealed a common metabolic pathway, glycerophospholipid metabolism associated with dulaglutide or liraglutide treatment. At 12 weeks of follow-up, the levels of lysoPCs were partially attenuated by dulaglutide. LysoPC can also be transformed into lysophosphatidic acid, sphingosine-1-phosphate, and other extracellular signaling lipids by plasma lysophospholipase D/autotoxin (34). A previous study has reported that the serum levels of lysoPC and lysoPE, which were associated with higher risk of cardiovascular diseases, were decreased in T2D patients compared to controls (35). Circulating lysoPCs have been identified as markers of a metabolically benign nonalcoholic fatty liver, suggesting that lysoPCs may play an important role in the pathophysiology of nonalcoholic fatty liver disease and its progression (36). The patterns observed in these metabolites indicate alterations in glycerophospholipid metabolism by dulaglutide that could be involved in the mechanisms linking GLP-1RAs action to a beneficial role in the cardiovascular system and attenuation of nonalcoholic steatohepatitis (NASH). Even though GLP-1R agonists are beneficial for modifying risk factors such as body weight, blood pressure, and lipids, these changes over time do not fully explain their effects on the reduction in MACE. Currently, there is no clear understanding of how GLP-1R agonists attenuate NASH, and whether glycerophospholipids are indicative of the protective effect in liver should be further determined.

In **Table 3**, these proteins seem to be potential targets of dulaglutide and liraglutide. In terms of hypoglycemic effect and improvement in pancreatic function, dulaglutide on a weekly basis is not inferior to liraglutide daily. However, the metabolic pathways involved in the pathological mechanism of dulaglutide and liraglutide were distinct. The insulin resistance signaling pathway was enriched in both the DU and LIGA groups (**Figures 5C, D**). We have illustrated the possible molecular mechanisms involved in GLP-1 dependent insulin sensitivity, including the amelioration of oxidative stress, inflammatory responses, plasma lipid profile, and the induction of insulin signaling pathways (37–40). Unexpectedly, the neuroactive ligand-receptor interaction signaling pathway was enriched in the DU group, while several neurotransmitter related signals such as the GABAergic synapse, dopaminergic synapse, and glutamatergic synapse were enriched in LIGA group. *In vitro* and *in vivo* studies using GLP-1RAs have demonstrated the neurotrophic and neuroprotective effects of GLP-1 receptor stimulation (41–43). In addition, adrenergic signaling in cardiomyocyte signaling and p53 signaling pathway were significantly related in the LIGA group. Impressively, the roles of the β-adrenergic receptor-mediated signaling pathway and the p53-mediated signaling pathway in the apoptosis of cardiomyocytes have been reported as major factors in the pathogenesis of heart failure (44). Hence, the reduction in non-fatal myocardial infarction detected with liraglutide is unlikely to be explained solely by GLP-1 receptor-mediated actions; A better understanding of how GLP-1R activation reduces the risk of myocardial infarction and cardiovascular death is needed. GLP-1RAs may target these proteins, as shown in **Table 3**. GLP1R, GNAS, and GCG were related to insulin resistance, which were verified by the previous studies (45). Recently, Yulia A et al. have reviewed recent progress and discoveries of T2D pharmacogenetics, including GLP-1RAs, and found that *GLP1R*, *TCF7L2*, *CNR1*, *SORCS1* and *WFS1* are associated with therapeutic responses to GLP1-RAs. Several candidate gene studies have been conducted to assess the role of GLP1R gene variants in the efficiency of GLP-1RAs treatment (46–48). The findings indicate that the different mechanisms underlined in the effects of GLP-1RAs used for T2D treatment are likely multifactorial. Although the proteins with high degrees of nodes may not represent the significantly altered proteins between the DU and LIGA groups, they might provide some insight into the underlying metabolism.

This study has three main strengths. First, since metabolic remodeling has been investigated in a well-characterized cohort with recent-onset T2D (49), our study was the first to compare two GLP-1RAs in metabolic remodeling in addition to the results of glycemic and extra-glycemic effectiveness. Second, we traced and profiled metabolic changes within individuals and performed a longitudinal analysis between two different long-acting GLP-1RAs. We found that glycerophospholipid remodeling acts as a metabolomic signature of dulaglutide and liraglutide. Third, the broader spectrum of glycerophospholipids in the cohort study allowed us to identify novel biomarkers and to uncover comprehensively the relationships of glycerophospholipids with GLP-1RAs and associated metabolic pathways, as a means of supporting the development of stratified and precision medicine.

There were several limitations to this study. First, the metabolic study had a relatively small sample size. Although the DU group was matched in age, sex, BMI, and key clinical indicators with the LIGA group, unmeasured factors such as dietary habits, medication, or supplements may also contribute. Second, the duration of 12 weeks of follow-up, although like that of phase III randomized controlled studies, was still relatively short. After 12 weeks of treatment, there was no clinically significant difference in body weight between the DU and LIGA groups. One potential explanation is that the duration of GLP-1RAs and the relatively small sample size could play a role, especially in the LIGA group. Finally, the DU group received the same guidance (low-calorie diet) from a dietitian as the LIGA group according to the clinical guideline of T2D. However, we did not collect data on diet composition and exercise record during the treatments.

## Data availability statement

The original contributions presented in the study are included in the article/**Supplementary Material**. Further inquiries can be directed to the corresponding authors.

## Ethics statement

The studies involving human participants were reviewed and approved by the Internal Review and Ethics Boards of Tongren Hospital, which is affiliated with Shanghai Jiao Tong University. The patients/participants provided their written informed consent to participate in this study.

## Author contributions

Studies were designed by XRG, XW and SH. JD wrote the manuscript. And ZZ did the metabolomics study. Sample collection was performed by LX, XXG, WYL, WP, XJ, WL, NZ. Data was analyzed by JD and ZZ. All authors contributed to the article and approved the submitted version.
