# Supplementary material for: Metabolic remodeling of glycerophospholipids acts as a signature of dulaglutide and liraglutide treatment in recent-onset type 2 diabetes mellitus
Source: Front Endocrinol (Lausanne). 2023 Jan 4;13:1097612. doi: 10.3389/fendo.2022.1097612 (PMC9846071; doi:10.3389/fendo.2022.1097612)
Supplement: Supplementary file 1 [file Table_1.docx]

**Supplementary Tables.**

| **Name** | **CTL group, N=28** | **T2D group, N=52** | **P value** |
| --- | --- | --- | --- |
| Age | 41 (35.25,25.50) | 47(37,58) | 0.232 |
| Sex male, n (%) | 17(60.70) | 33 (63.50) | 0.810 |
| Weight, kg | 62.55(56.80,71.88) | 82.5(72.85,92.25) | 0.000 |
| BMI, kg/m2 | 22.64±1.29 | 28.78±3.92 | 0.000 |
| SBP, mmHg | 125(118,130) | 136(125.5,149.25) | 0.000 |
| DBP, mmHg | 79.43±6.09 | 87.19±13.06 | 0.003 |
| FPG, mg/dl | 5(4.8,5.48) | 8.2(6.83,9.9) | 0.000 |
| HbA1c, % | 5.45(5.3,5.58) | 9.55(8.23,11.1) | 0.000 |
| TC, mg/dl | 3.89±0.7 | 4.97±1.12 | 0.003 |
| HDL cholesterol, mg/dl | 1.03(0.96,1.24) | 0.91(0.83,1.11) | 0.007 |
| Triglycerides, mg/dl | 1.12±0.36 | 2.14±0.97 | 0.000 |
| LDL cholesterol, mg/dl | 2.3±0.69 | 3.47±1.22 | 0.001 |
| AST, U/l | 20.5(18,27.5) | 26.5(20.25,38.75) | 0.007 |
| ALT, U/l | 23(18.5,28) | 39.5(24,61) | 0.000 |
| eGFR, ml/min/1.73 m^2^ | 116.95±9.25 | 122.28±9.25 | 0.001 |

**Table S1. The characteristics of control and diabetes patients**

Abbreviations: BMI, body mass index; SBP, systolic blood pressure; DBP, diastolic blood pressure; FBG, fasting blood glucose; HbA1c, glycated hemoglobin; TG, triglycerides; TC, total cholesterol; LDL-c, low density lipoprotein cholesterol; HDL-cholesterol, high density lipoprotein cholesterol. ALT, alanine transaminase; AST, aspartate aminotransferase; eGFR, estimated glomerular filtration rate. Data are presented as Min to Max. Differences between the T2D group and the CTL group were tested by independent sample t test (normally distributed variables) or Mann-Whitney U (variables with skewed distribution) test. *p<0.05, **p<0.01.

**Table S2. Top-ranking differentially regulated metabolites in serum at baseline between the CTL and T2D groups^#^.**

| **Name** | **Class** | **log2 Fold Change** | ***P*value** |
| --- | --- | --- | --- |
| PE(16:0/0:0) | Glycerophospholipids | 1.974 | ＜0.001 |
| GlcCer(d16:1/23:0) | Sphingolipids | 1.987 | ＜0.001 |
| PI(22:2(13Z,16Z)/22:6(4Z,7Z,10Z,13Z,16Z,19Z)) | Glycerophospholipids | 2.072 | ＜0.001 |
| PG(21:0/22:4(7Z,10Z,13Z,16Z)) | Glycerophospholipids | 2.118 | ＜0.001 |
| PC(18:3(6Z,9Z,12Z)/22:6(4Z,7Z,10Z,13Z,16Z,19Z)) | Glycerophospholipids | 2.134 | ＜0.001 |
| Ferrioxamine B | Fatty Acyls | 2.191 | ＜0.001 |
| PC(14:0/22:5(4Z,7Z,10Z,13Z,16Z)) | Glycerophospholipids | 2.198 | ＜0.001 |
| Neuromedin N | Carboxylic acids and derivatives | 2.274 | ＜0.001 |
| alpha-Chaconine | Steroids and steroid derivatives | 2.382 | ＜0.001 |
| Nicotinamide | Pyridines and derivatives | 2.459 | ＜0.001 |
| L-Isoleucine | Carboxylic acids and derivatives | 2.476 | ＜0.001 |
| SM(d18:2/24:1) | Sphingolipids | 2.752 | ＜0.001 |
| PI(19:1(9Z)/0:0) | Glycerophospholipids | 2.802 | ＜0.001 |
| PS(17:0/22:2(13Z,16Z)) | Glycerophospholipids | 2.819 | 0.004 |
| Glycine | Carboxylic acids and derivatives | 2.872 | ＜0.001 |
| PS(O-16:0/19:1(9Z)) | Glycerophospholipids | 2.878 | ＜0.001 |
| PS(15:0/22:0) | Glycerophospholipids | 2.979 | 0.003 |
| SM(d18:0/22:1(13Z)) | Sphingolipids | 3.041 | ＜0.001 |
| (+/-)11-HETE | Fatty Acyls | 3.220 | ＜0.001 |
| PG(16:0/22:1(11Z)) | Glycerophospholipids | 3.482 | 0.005 |
| Ethylenediaminetetraacetic acid | Carboxylic acids and derivatives | -11.014 | ＜0.001 |
| D-Mannitol | Organooxygen compounds | -7.069 | ＜0.001 |
| Galactitol | Organooxygen compounds | -6.395 | ＜0.001 |
| 2,3-Dimethylsuccinic acid | Fatty Acyls | -4.820 | ＜0.001 |
| Citric acid | Carboxylic acids and derivatives | -4.260 | ＜0.001 |
| PC(O-18:2(9Z,12Z)/2:0) | Glycerophospholipids | -4.220 | ＜0.001 |
| N-Acetylglutamine | Carboxylic acids and derivatives | -3.771 | ＜0.001 |
| Lyso-PAF C-16 | Glycerophospholipids | -3.704 | ＜0.001 |
| D-α-Tocopherol | Prenol lipids | -3.619 | ＜0.001 |
| LysoPC(P-18:0/0:0) | Glycerophospholipids | -3.618 | ＜0.001 |
| trans-4-Hydroxy-L-proline | Carboxylic acids and derivatives | -3.265 | ＜0.001 |
| 2-Oxoglutaric acid | Keto acids and derivatives | -3.141 | ＜0.001 |
| D-Erythro-sphingosine 1-phosphate | Sphingolipids | -3.010 | ＜0.001 |
| L-isoglutamine | Carboxylic acids and derivatives | -2.966 | ＜0.001 |
| Glutaconic acid | Carboxylic acids and derivatives | -2.809 | ＜0.001 |
| LysoPC(18:2(9Z,12Z)/0:0) | Glycerophospholipids | -2.791 | ＜0.001 |
| Cytidine 5'-diphosphocholine | Pyrimidine nucleotides | -2.770 | ＜0.001 |
| PC(O-18:1(10E)/2:0) | Glycerophospholipids | -2.671 | ＜0.001 |
| LysoPC(18:3(6Z,9Z,12Z)/0:0) | Glycerophospholipids | -2.598 | ＜0.001 |
| PC(22:4(7Z,10Z,13Z,16Z)/22:5(4Z,7Z,10Z,13Z,16Z)) | Glycerophospholipids | -2.575 | ＜0.001 |

^#^ The table includes top-25 up-regulated and down-regulated metabolites with p value＜0.05 (2-sided t-test). **Supplementary Figures.**

**
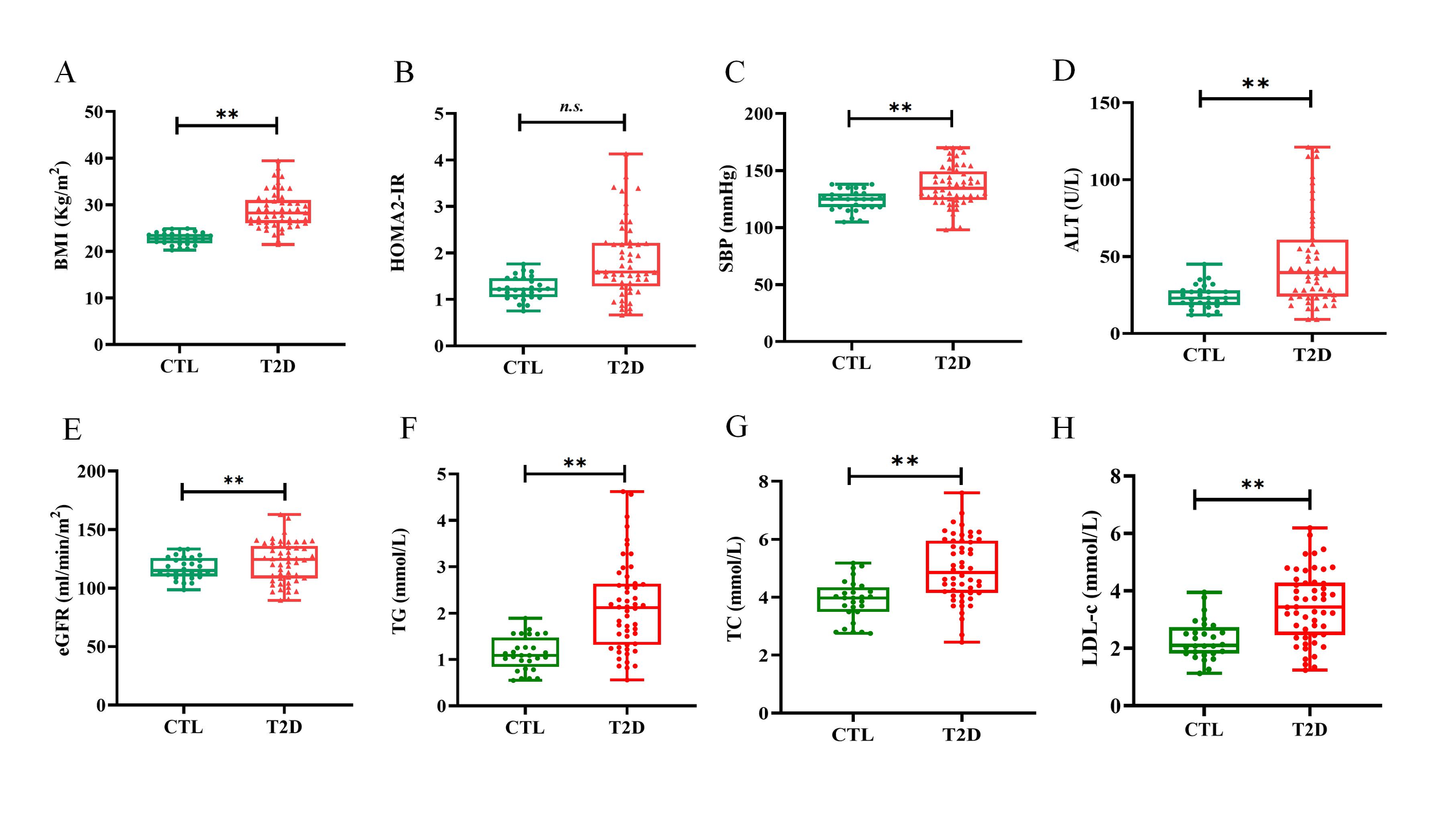
**

**Figure S1. General characteristics of participants at baseline in the CTL and T2D groups.** Abbreviations: BMI, body mass index; SBP, systolic blood pressure; DBP, diastolic blood pressure; ALT, alanine transaminase; eGFR, estimated glomerular filtration rate; TG, triglycerides; TC, total cholesterol; LDL-c, low density lipoprotein cholesterol. Data are presented as Min to Max. Differences between the T2D group and the CTL group were tested by independent sample t test (normally distributed variables) or Mann–Whitney U (variables with skewed distribution) test. **p<0.01.


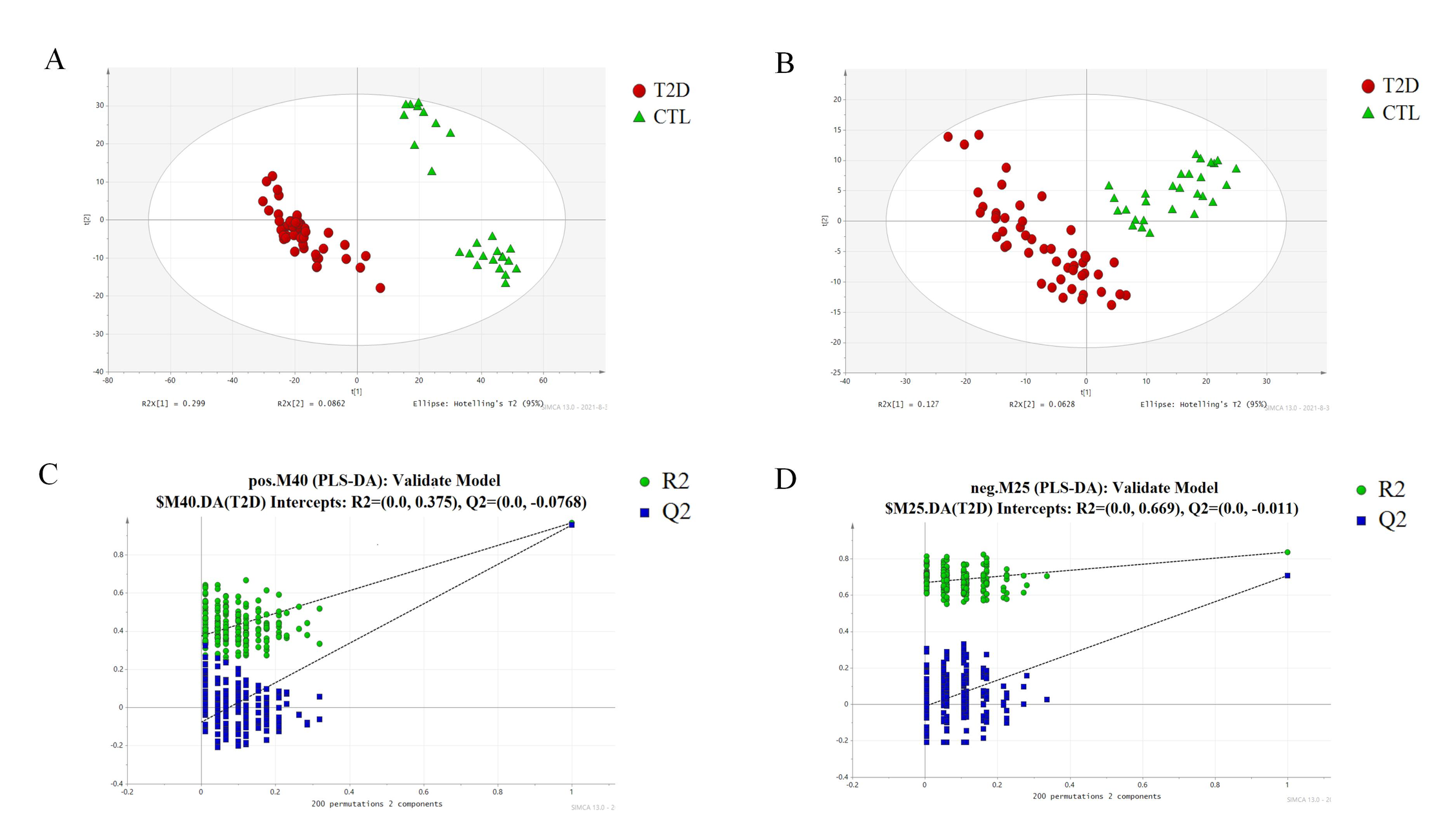


**Figure S2. The score plot of the PLS-DA model.** The score plot of the PLS-DA model in the positive mode (A) and the negative mode (B). Permutation test of the PLS-DA model in the positive mode (C) and the negative mode (D); Green dots indicate R2 and blue dots indicate Q2.

**
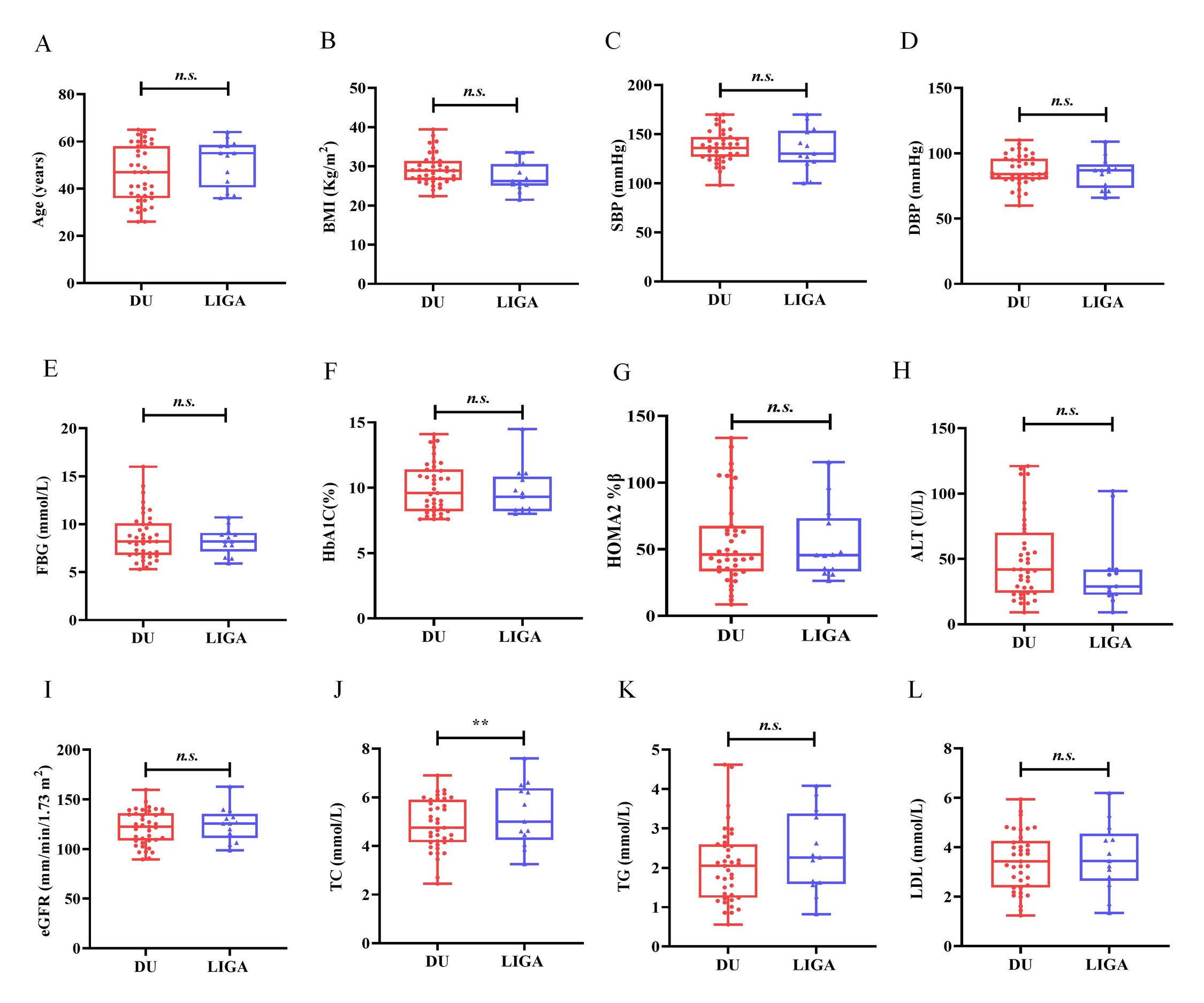
Figure S3. Baseline characteristics of participants in the DU and LIGA groups.** The levels of age (A), BMI (B), SBP (C), DBP (D), FBG (E), HbA1c (F) and HOMA2 %β (G), ALT (H), eGFR (I), TC (J), TG (K) and LDL (L) in the DU (n = 39) and LIGA groups (n = 13). Abbreviations: BMI, body mass index; FBG, fasting blood glucose; HbA1c, glycated hemoglobin; HOMA2 β%, homeostasis model assessment of beta cell function index; HOMA-IR homeostatic model assessment of insulin resistance; SBP, systolic blood pressure; DBP, diastolic blood pressure; ALT, alanine transaminase; eGFR, estimated glomerular filtration rate; TG, triglycerides, TC, total cholesterol; LDL, low density lipoprotein. Data are presented as Min to Max. Differences between the DU group and the LIGA group were tested by independent sample t test (normally distributed variables) or Mann-Whitney U (variables with skewed distribution) test. ** p<0.01, *n.s.* the difference is not statistically significant.


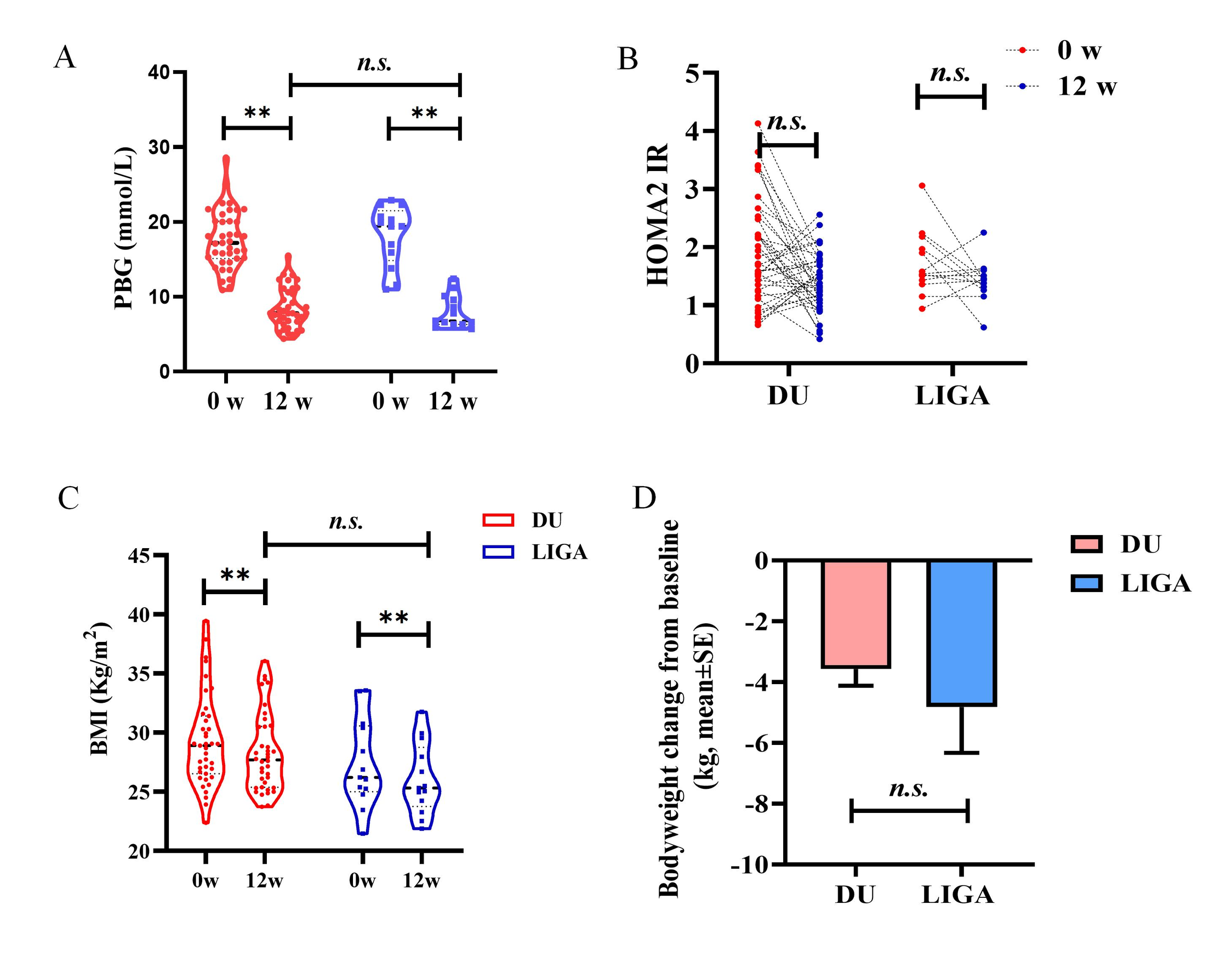


**Figure S4. Change in 2-hour postprandial blood glucose concentrations, homeostatic model assessment of insulin resistance, BMI and body weight from baseline to week 12 in DU and LIGA groups.** (A) Change in 2-hour postprandial blood glucose concentrations from the beginning to the end of follow-up (12^th^ week). (B) Change in homeostatic model assessment of insulin resistance from the beginning to the end of follow-up (12^th^ week). (C) Change in BMI from baseline to week 12. (D) Percent change in body weight from the beginning to the end of follow-up (12^th^ week). Abbreviations: PBG, OGTT-2h blood glucose; BMI, body mass index; HOMA-IR, homeostasis model assessment of beta cell function index. ** p<0.01; *n.s.* the difference is not statistically significant.


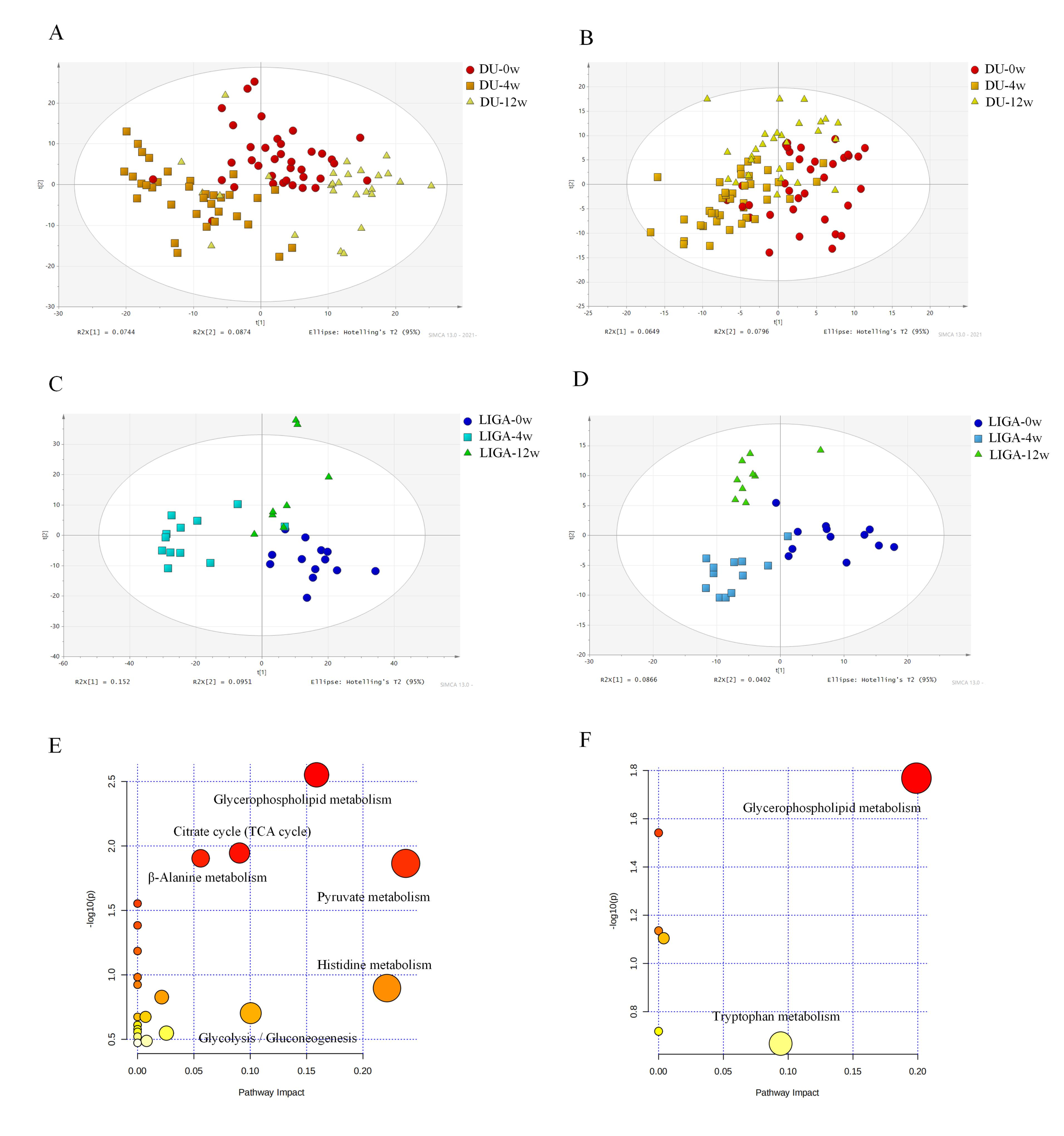


**Figure S5. PLS-DA model and metabolic pathways undergoing significant changes from baseline to 12-week GLP-1RAs treatment.** (A, B) PLS-DA model comparing metabolites from the beginning to 12-week dulaglutide in the positive mode and the negative mode. (C, D) PLS-DA model comparing metabolites from the beginning to 12-week liraglutide in the positive mode and the negative mode. A score plot showing the class-discriminatory component 1 (x-axis) versus class-discriminatory component 2 (y-axis). (E) Metabolic pathways undergoing significant changes during DU treatment. (F) Metabolic pathways undergoing significant changes during LIGA treatment.


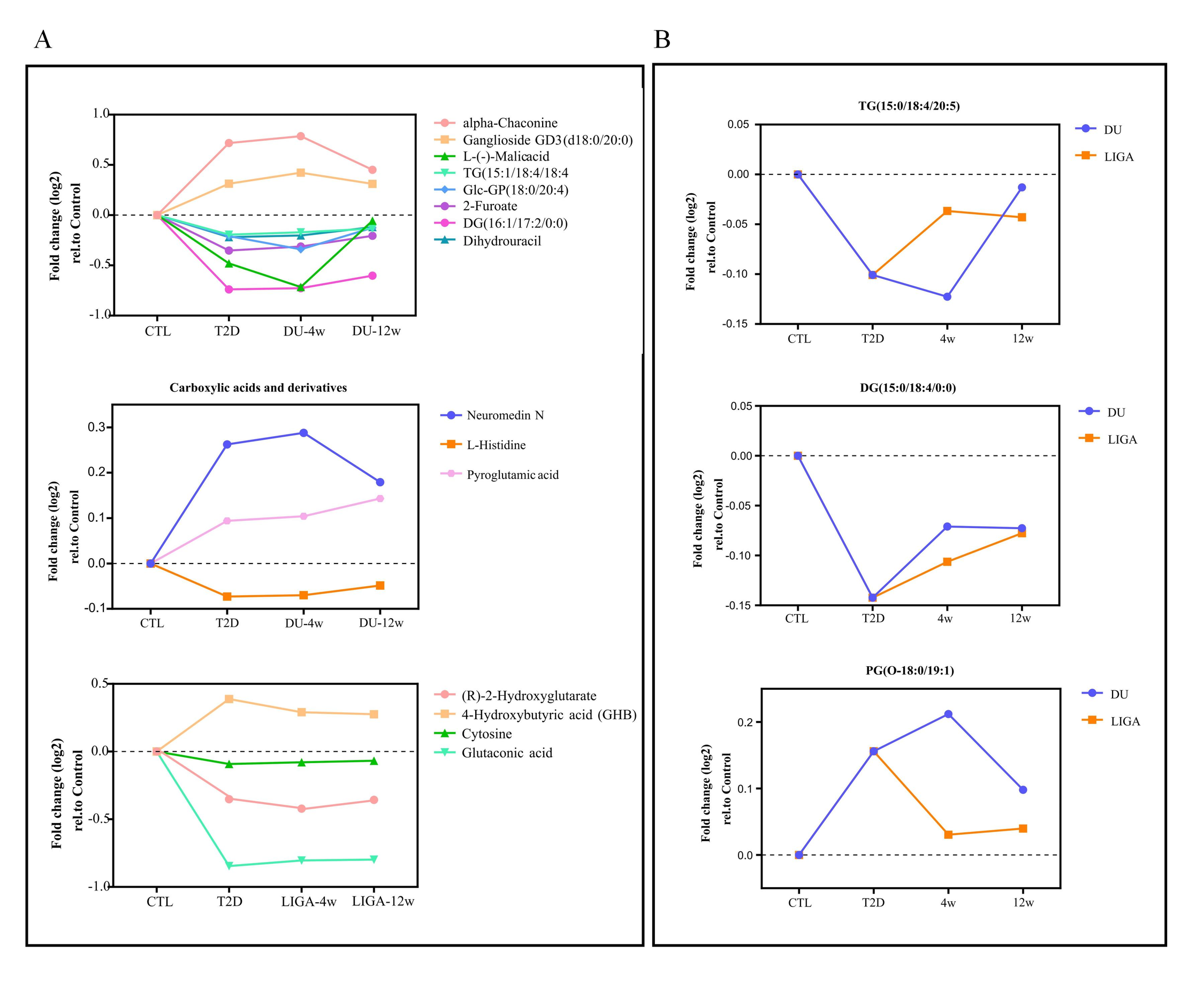


**Figure S6. Other metabolite groups altered during DU and LIGA treatment apart from glycerophospholipid.** (A) Dynamic changes of differential metabolites at week 4 and week 12 both in response to DU and LIGA. (B) There metabolites were commonly regulated both in DU and LIGA group, including PG(O-18:0/19:1, TG(15:0/18:4/20:5 and PG(O-18:0/19:1). The dots represent the mean log^2^ fold change relative to control group.
